# Immunohistochemical Expression of Human Epidermal Growth Factor Receptor 2 and Ki67 in Apocrine Gland Anal Sac Adenocarcinoma

**DOI:** 10.3390/ijms25126451

**Published:** 2024-06-12

**Authors:** Felipe Paiva, Júlio Santos, Gabriel Carra, Felipe Sueiro, Paulo Jark, Andrigo Nardi

**Affiliations:** 1 Department of Veterinary Clinic and Surgery, Universidade Estadual Paulista (UNESP), Jaboticabal 14884-900, Brazil; 2 School of Agrarian Studies, Goyazes University Center (UNIGOYAZES), Trindade 75393-365, Brazil; 3 Histopathological Diagnosis Department, VETPAT—Animal Pathology & Molecular Biology, Campinas 13073-022, Brazil; 4 Clinical Oncology, Onccarevet e Onconnectionvet, Ribeirão Preto 14026-587, Brazil

**Keywords:** anal sac apocrine adenocarcinoma, immunohistochemistry, neoplasia, prognosis, therapeutic target

## Abstract

Apocrine gland anal sac adenocarcinoma is an aggressive neoplasm, and surgery remains the treatment of choice, although it is controversial in advanced cases. The prognostic factors are not well established. Human Epidermal Growth Factor Receptor 2 (HER2) is a membrane protein related to tumorigenesis, whereas Ki67 is a nuclear protein related to cell proliferation. Both are potential prognostic markers and therapeutic targets. This study aimed to evaluate the expression of HER2 and Ki67 markers in canine apocrine gland anal sac adenocarcinoma. The tumor samples were divided into four groups: largest tumor diameter less than 2.5 cm, largest tumor diameter greater than 2.5 cm, metastatic lymph nodes, and control group of non-neoplastic anal sacs. Each contained 10 samples. Immunohistochemistry was performed to verify the expression of HER2 and Ki67 markers. Positive HER2 staining was observed in 45% of the neoplastic cases and negative HER2 staining in 100% of the control group. The Ki67 expression had a median of 25% in all groups, except for the control group, which had a median of 8%. The HER2 and Ki67 expression was present in apocrine gland anal sac adenocarcinoma, making them potential therapeutic targets. However, it was not possible to determine the clinical value of either marker.

## 1. Introduction

Apocrine gland anal sac adenocarcinoma (AGASACA) originates from the apocrine glands, located in the anal sac [1,2]. It accounts for 17% of tumors in the perianal region and 2% of all cutaneous neoplasms in dogs [1,2]. The tumor begins faintly, intradermally, with subcutaneous masses only perceptible on rectal palpation, whereas advanced cases involve large protuberant masses with varied aspects [1,2,3]. The behavior of neoplasms has variable local aggressiveness, with high metastatic potential. About 26–96% of them evolve to local metastasis and 0–42% to distant metastasis from the time of initial diagnosis [1,2,3].

The treatment of choice is surgical excision, which is considered effective in smaller tumors but complex in advanced cases [2,3]. Adjuvant modalities can be applied, including chemotherapy, radiotherapy, electrochemotherapy, molecular targeted therapy, and use of cyclooxygenase-2 inhibitors. However, these modalities still need further confirmation regarding their effectiveness [2,4,5,6]. The prognostic factors include clinical staging, tumor diameter, presence of regional and distant metastases, presence of paraneoplastic hypercalcemia, and histological characteristics of the tumor [1,2,7]. Prognosis ranges from reserved to unfavorable, with a survival time between 0 and 1873 days [1,2,3]. In this context, the validation of new predictive and prognostic markers, as well as the search for new therapeutic targets, is essential.

HER2 is a membrane protein belonging to the epidermal growth factor receptor (EGFR/HER) family, with normal expression in organic tissues and functions related to cell growth and differentiation. When overexpressed, the HER2 receptor triggers proliferative stimuli in addition to the deregulation of cell control mechanisms, promoting tumorigenesis [8,9,10]. The presence of HER2 receptors has been proven to increase in several neoplastic types in humans and animals [8,10,11,12,13,14,15,16,17]. Its potential as a therapeutic target has been investigated using anti-HER2 monoclonal antibodies [18,19,20,21] as well as anti-EGFR/HER2 polyclonal and recombinant listeria vaccines [22,23,24].

Ki67 is a nuclear protein with functions related to cell proliferation [25]. It is present in cells active in the cell cycle while being minimal or absent in quiescent cells [26,27]. Its expression is observed in all proliferating cells, normal or neoplastic, associated with a short half-life of approximately 1 h, and regulated by a balanced mechanism of protein syn-thesis and degradation [1,27,28]. Due to its metabolic and functional characteristics, Ki67 is used as one of the main markers of cell proliferation, widely used in human and veterinary oncology [1,25]. Its application as a prognostic marker of the proliferative index has been described for different tumor types in dogs [1,2].

This study evaluates the expression of the HER2 and Ki67 markers in cases of canine AGASACA regarding their presence and clinical value.

## 2. Results

Analysis of the expression of the HER2 marker in the primary anal sac tumor samples revealed positive and negative staining in 45% and 55% of the cases, respectively. The markings observed in each group are presented in Table 1.

**Figure 1:**
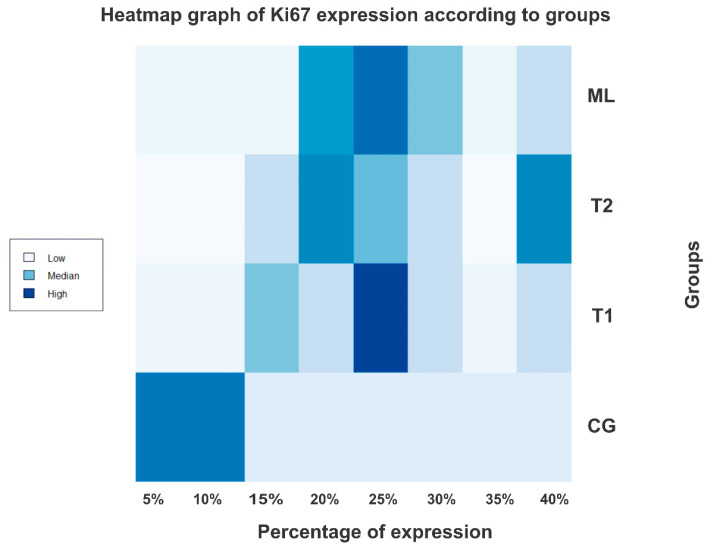
Heatmap graphic of the Ki67 expression according to groups. CG (control group), T1 (largest tumor diameter smaller than 2.5 cm), T2 (largest tumor diameter larger than 2.5 cm), and ML (metastatic lymph node group).

In the analysis of Ki67 marker expression, the following results were obtained in the groups: T1, median of 25% and mean of 25% (ranging from 15% to 40%); T2, median of 25% and mean of 28% (ranging from 15% to 40%); ML, median of 25% and mean of 26% (ranging from 20% to 40%); and CG, median of 8% and mean of 8% (ranging from 5% to 10%). The values observed in each group are presented in Table 2 and illustrated in Figure 1.

The Kruskal–Wallis chi-square test was used to verify the difference in the HER2 expression between the CG, T1, and T2 groups; this result was considered significant as the CG differed from the other groups (*p* = 0.036) despite the absence of difference between T1 and T2 groups (*p* = 0.500). Using the same test to verify the association between Ki67 markings and the CG, T1, and T2 groups, no difference was observed between the groups (*p* = 0.460).

Cohen’s kappa coefficient test showed a minimal agreement (K = 0.333, *p* = 0.05) of Ki67 expression in primary tumor samples and their corresponding metastatic lymph node, whereas in the HER2 expression, no significant agreement was observed, due to the lack of confidence (K = 0.400, *p* = 0.197).

## 3. Discussion

Immunohistochemistry revealed positive HER2 staining in 45% of the neoplastic cases and negative HER2 staining in 100% of the control group. The Ki67 expression had a median of 25% in all groups, except for the control group, which had a median of 8%.

Several studies have investigated tumor size as a prognostic factor in cases of AGASACA, related to different cutoff values [3,29,30]. The size suggested by Polton and Brearley (2007) [31] in their staging model is the most accepted, with the measurement performed through clinical evaluation with a caliper. However, in the present study, the tumor size measurement was performed directly on formalin-fixed tissue. The concordance between the different measurement methods has previously been evaluated, showing moderate concordance between the methods of clinical evaluation of the tumor mass and evaluation of formalin-fixed tissue [30].

The HER2 expression has already been investigated in veterinary medicine in different tumor types. Tsuboi et al. (2019) [12] evaluated the expression related to urothelial carcinomas in the bladder and found positive staining in 60.9% of carcinoma cases, 37.5% of polypoid cystitis cases, and 0% in normal bladder tissue. Furthermore, Yoshimoto et al. (2019) [11] evaluated its expression in thyroid carcinomas, with positive staining in 48% of cases, whereas in a second study, Yoshimoto et al. (2020) [15] evaluated its expression in primary lung tumors, with positive staining in 69% of cases. Sakai et al. (2021) [32] evaluated its expression in prostatic carcinoma and found 100% negative staining in normal prostate tissue and positive staining in 61.5% of prostatic carcinoma cases.

Brunetti et al. (2021) [33] conducted a broader study, evaluating carcinomas of different origins, including cutaneous, oral squamous cell, gastrointestinal, rectal, pulmonary, urothelial, prostatic, and ovarian carcinomas. They found higher expression mainly in carcinomas originating from the intestinal tract, but with markings also present in all other types evaluated. The recent interest in identifying HER2 expression in canine tumors is related to its potential as a therapeutic target, with advances in molecular targeted therapy directed at HER2 receptors, through receptor inhibitors and antiEGFR/HER tumor vaccines [18,19,20,21,22,23,24].

The HER2 expression in AGASACAs has also been previously described by Yoshimoto et al. (2019) [17], who evaluated 25 AGASACA cases via immunohistochemistry, like the present study, with positive markings in 80% of cases and a predominance of cases with a score of 2+ (56%), and with a smaller number of cases with a score of 3+ (24%). In the present study, when evaluating the expression in samples from the primary tumor with the addition of T1 and T2, we found positive staining in 45% of the cases, represented only by cases with a score of 2+, with no case with a score of 3+. Despite the numerically lower labeling, the role of HER2 in this neoplastic type cannot be ruled out, especially considering the statistical significance, confirmed by the chi-squared test, in relation to the absence of labeling in the intact anal sac samples.

Previously, only tumors with strong HER2 expression were considered for specific targeted therapies. However, recent studies have introduced the “HER2-low” category, which is currently described in human breast carcinomas, and the efficacy of several therapeutic compounds has been proven in this category [34,35,36]. In this sense, this finding is even more important due to the possibility of specific treatment even in cases with lower scores.

The statistical tests revealed no association between the HER2 marker expression and tumor size, with no difference between the T1 and T2 groups. This finding indicates that the expression is present even in tumors with smaller dimensions, diagnosed in the early stages, demonstrating that even these cases can benefit from targeted therapies. In the present study, the HER2 expression was also observed in samples from ML nodes, with no statistical difference in expression in relation to primary tumors, making it impossible to evaluate its value as a prognostic marker. Previous studies have not evaluated its expression in samples of ML nodes but have attempted to correlate this marker with other clinical factors, finding no apparent correlation [11,12,15,17,32].

Regarding protein Ki67, its prognostic role in the evaluation of different tumor types, and its positive markings in AGASACAs, has already been proven [1,2,37,38]; however, its expression in quantitative evaluation has shown varied results. In the present study, the median, mean, and variation observed in the T1, T2, and ML groups are similar to the values described by Skorupski et al. (2018) [29], who observed a median of 25% and a mean of 26% (ranging from 13% to 48%). Other studies have demonstrated higher values, with a median of 34.33% and mean of 34.58% (ranging from 19.6% to 55.98%) [39], or lower values, with a median of 7.75% (ranging from 0% to 54%) [40]. Given the conflicting findings, it was impossible to assess the prognostic value or suggest a cutoff value associated with the expression of this marker; thus, further studies are required for this purpose.

The concordance of expression between primary tumors and ML nodes, as well as the association with tumor size, was not statistically proven in this study. The association of Ki67 markings with other clinical factors could also not be proven in similar studies [32,39,40].

## 4. Materials and Methods

### 4.1. Samples and Groups

A total of 20 cases of canine AGASACA were selected, with histopathological confirmation from a partner laboratory. Of the cases, 10 had a largest tumor diameter smaller than 2.5 cm, composing the T1 group, and 10 had a largest tumor diameter greater than 2.5 cm, composing the T2 group. The cutoff point of 2.5 cm was determined according to the tumor staging model proposed by Polton and Brearley (2007) [31]. Tumor size measurement was performed on the surgical specimen, at the time the sample entered the laboratory, considering only the neoformation present in the total sample received.

Among the 20 initially selected cases, 10 also had metastatic lymph node involvement. Samples of these lymph nodes were also selected, composing the metastatic lymph node (ML) group. In addition, 10 samples of intact anal sacs were collected from a partner veterinary hospital. These samples belonged to animals whose death was not related to neoplastic disease, composing the control group (CG).

Each of the 40 samples (10 from each group) was paraffin-embedded and subsequently submitted to immunohistochemical evaluation to verify the expression of HER2 and Ki67 markers.

### 4.2. Immunohistochemistry

Immunohistochemistry reactions were performed at the partner laboratory and evaluated by a dedicated pathologist with expertise in scoring HER2. The fragments previously embedded in paraffin blocks were cut to a 4 μm thickness, placed on previously marked slides for microscopy, and subsequently deparaffinized, rehydrated, and washed with phosphate-buffered solution (PBS). Endogenous peroxidase was then blocked, followed by washing with PBS and antigenic recovery by humid heat in an EDTA (pH 8.9) in a Pascal–Dako pressure cooker. Finally, the slides were cooled to room temperature and washed with Tris-buffered solution.

Next, the primary antibody was applied to cover the entire fragment, and the slides were transferred to an oven at 37 °C for 40 min and then stored in a refrigerator at 4 °C for 16 h (overnight). Subsequently, the slides were washed with PBS, the secondary antibody was applied, and the slides were placed in an oven at 37 °C for 35 min. Then, the slides were washed with PBS, and chromogen 3,3′-diaminobendizidine was applied and kept on the sections for up to 5 min.

Finally, counterstaining with Harris hematoxylin was performed, followed by passage in ammonia and washing of the slides in running water. The slides were then diaphonized and mounted. Evaluation of the immunohistochemistry results was conducted under optical light microscopy, with a 40× objective.

The antibodies used were C-erbB-2 Oncoprotein (Concentrate) (Clone Polyclonal, Code Number A0485, DakoCyto-mation, Carpinteria, CA, USA) and Ki-67 Antigen (Clone MIB-1, Code Number M7240, DakoCyto-mation, Carpinteria, CA, USA). Positive and negative controls were used.

**Figure 2:**
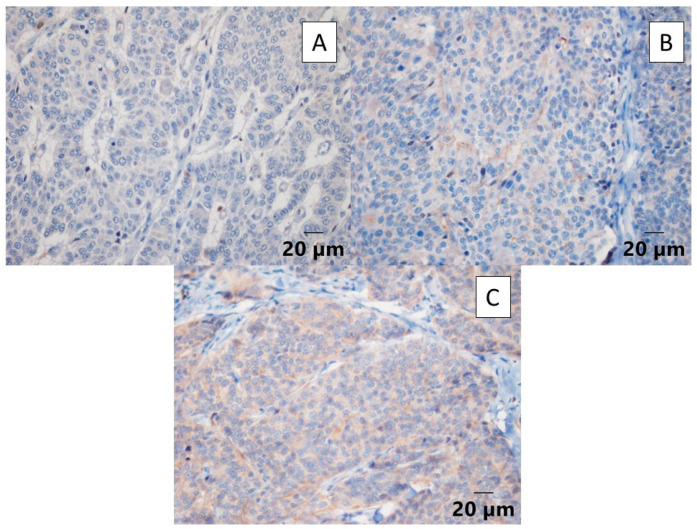
Photomicrographs of human epidermal growth factor receptor 2 (HER2) immunostaining. (**A**) Immunostaining in AGASACA with a score of 0 (40× magnification). (**B**) Immunostaining in AGASACA with a score of 1+ (40× magnification). (**C**) Immunostaining in AGASACA with a score of 2+ (40× magnification).

Evaluation of the HER2 expression was conducted using a scoring system based on the guidelines proposed by the American Society of Clinical Oncology (ASCO) and the College of American Clinical Pathologists (CAP) [41,42], as presented in Table 3. For standardization purposes, scores 2+ and 3+ were considered as positive and scores 0 and 1+ as negative. Figure 2 presents the markings 3+, 2+, and 1+.

Evaluation of the Ki67 expression was determined by the percentage of positive nuclei in at least 500 neoplastic cells, assessed in at least eight randomly selected fields representative of the Ki67 range [27]. Each nucleus that exhibited evidence of expression was considered positive for the Ki67 expression.

### 4.3. Statistical Analysis

Statistical analysis was conducted using the statistical package IBM SPSS Statistics version 25 (IBM^®^, New York, NY, USA), and a 95% confidence interval was used for all tests (*p* ≤ 0.05). Fisher’s exact test was conducted to verify whether there was an association between the HER2 expression in tumors and lymph nodes according to different sizes. Furthermore, the chi-squared test was performed to verify the association regarding Ki67. A kappa test was also conducted to determine if there was any agreement between the HER2 and Ki67 expressions of the tumors and their respective metastatic lymph nodes.

### 4.4. Study Limitations

This study is subject to inherent limitations, including its retrospective design, potential biases, and a limited sample size. The retrospective nature of this study may constrain causal inference, and the small sample size may impact generalizability. Despite these constraints, rigorous measures were taken to mitigate biases.

## 5. Conclusions

In conclusion, the HER2 expression was present in AGASACAs, even in those with small dimensions, defined as having a largest tumor diameter smaller than 2.5 cm, as well as in regional lymph nodes with established metastasis. The expression observed, in 45% of the cases, was lower than that demonstrated in other studies. However, when compared with the expression in intact anal sacs, in 0% of the cases, it is important in the neoplastic process. We can also conclude that AGASACAs demonstrably express the Ki67 protein, although its real prognostic value remains uncertain. Both markers exhibited clear expression and are potential therapeutic targets for the ongoing development of new drugs.

## Figures and Tables

**Table 1:** HER2 expression described in scores according to established groups.

	SCORE	Score 0	Score 1+	Score 2+	Score 3+
GROUP		Negative Staining		Positive Staining	
CG (N = 10)		10/10 (100%)	0	0	0
T1 (N = 10)		1/10 (10%)	5/10 (50%)	4/10 (40%)	0
T2 (N = 10)		0	5/10 (50%)	5/10 (50%)	0
ML (N = 10)		0	6/10 (60%)	4/10 (40%)	0

Caption: CG (control group), T1 (largest tumor diameter smaller than 2.5 cm), T2 (largest tumor diameter larger than 2.5 cm), and ML (metastatic lymph node group).

**Table 2:** Ki67 expression described in percentage values according to the established groups.

	SCORE	5%	10%	15%	20%	25%	30%	35%	40%
GROUP									
CG (N = 10)		5/10 (50%)	5/10 (50%)	0	0	0	0	0	0
T1 (N = 10)		0	0	2/10 (20%)	1/10 (10%)	5/10 (50%)	1/10 (10%)	0	1/10 (10%)
T2 (N = 10)		0	0	1/10 (10%)	3/10 (30%)	2/10 (20%)	1/10 (10%)	0	3/10 (30%)
ML (N = 10)		0	0	0	3/10 (30%)	4/10 (40%)	2/10 (20%)	0	1/10 (10%)

Caption: CG (control group), T1 (largest tumor diameter smaller than 2.5 cm), T2 (largest tumor diameter larger than 2.5 cm), and ML (metastatic lymph node group).

**Table 3:** Classification model of the HER2 expression according to scores proposed by ASCO/CAP.

Score	Description
3+	Circumferential marking on the membrane that is complete, intense, with the presence of >10% of tumor cells.
2+	Circumferential staining on the membrane that is incomplete and/or weak/moderate, with presence of >10% of tumor cells; or circumferential staining on the membrane that is complete and intense, with the presence of ≤10% of tumor cells.
1+	Membrane labeling that is incomplete, very weak/almost unnoticeable, with >10% of tumor cells present.
0	No marking observed; or membrane labeling that is incomplete, very faint/almost unnoticeable, with ≤10% of tumor cells present.

## Data Availability

Data are contained within the article.
